# Mitochondrial small fiber neuropathy as a novel phenotypic trait of Leigh-like syndrome due to the variant m.10191T>C in *MT-ND3*

**DOI:** 10.1016/j.clinsp.2023.100206

**Published:** 2023-05-15

**Authors:** Shaundra M. Newstead, Carla A. Scorza, Ana C. Fiorini, Fulvio A. Scorza, Josef Finsterer

**Affiliations:** a HeatSync Biochemistry Laboratory, Mesa, United States; b Disciplina de Neurociência, Universidade Federal de São Paulo/Escola Paulista de Medicina (UNIFESP/EPM), São Paulo, SP, Brazil; c Programa de Estudos Pós-Graduado em Fonoaudiologia, Pontifícia Universidade Católica de São Paulo (PUC-SP), São Paulo, SP, Brazil; d Departamento de Fonoaudiologia, Escola Paulista de Medicina/Universidade Federal de São Paulo (EPM/UNIFESP), São Paulo, SP, Brazil; e Neurology and Neurophysiology Center, Vienna, Austria

The mitochondrial DNA (mtDNA) variant m.10191T>C in *MT-ND3* has been described in about 30 patients so far.^1^ In most of these cases, the variant presented phenotypically as Leigh syndrome or Leigh-like syndrome.^1^ Although Large Fiber Neuropathy (LFN) has been reported as part of the phenotype in some of these patients.^2^^,^^3^ Small Fiber Neuropathy (SFN) has not yet been reported.

The patient is a 32-year-old female who developed a progressive multi-organ syndrome manifesting in: the Central Nervous System (CNS) with epilepsy, choreoathetosis, cerebellar ataxia, migraine, mild cognitive impairment, micropsia, idiopathic intracranial hypertension, hypersomnia, central apnea, symmetric pallidal hypointensities, panda sign, and calcifications; the endocrine organs with empty sella syndrome, hypocorticism, hypoaldosteronism, and hypogonadism; the hematopoietic system with pancytopenia, pseudohemolytic anemia, and B-cell lymphocytosis; the immune system with hypogammaglulinemia and lymphadenopathy; the intestine with dysphagia, reflux, and chronic diarrhea; the kidneys with mild renal insufficiency, renal tubular acidosis, and nephrolithiasis; the muscle with myopathy, ptosis, diplopia, myalgia, exercise intolerance, easy fatigability, and lactic acidosis; and the connective tissue with hyperlaxity of joints, bruising, scoliosis, and Chiari malformation. In addition, she complained of recurrent syncope since the age of 7, regional, stabbing pain since age 18, and orthostatic hypotension since age 23. None of the clinically manifesting first-degree relatives presented symptoms of SFN.

Genetic processing at the age of 23 revealed the known, pathogenic variant m.10191T>C in *MT-ND3*. Cerebral MRI showed bilateral globus pallidus hypointensities and midbrain Panda sign. The cerebral CT additionally showed abnormal calcification high parietal. Work-up for stabbing pain and dysautonomia using Nerve Conduction Studies (NCSs) of the median, ulnar, peroneal, tibial, and sural nerves were uninformative. The tilt table test revealed Postural Tachycardia Syndrome (POTS). Skin biopsy at age 23 from the right upper thigh, right calf, and right forearm in Zamboni fixative and immune-stained with PGP9.5 yielded Intra-Epidermal Nerve fiber Densities (IENFD) of 3.4 (n > 7.0 mm), 6.5 (n > 8.4 mm), and 12.3 (n > 4.0 mm),^4^ confirming the suspected diagnosis of SFN. Work-up of secondary causes of SFN,^5^ including HbA1c, viral, bacterial, plasmodial, or fungal infections, Borreliosis, connective tissue disease, autoimmune disorder, anti-neuronal antibodies, malignancy, monoclonal gammopathy, vaccinations, various toxins, and critical illness was uninformative. Since creatinine was only slightly elevated, SFN was interpreted as a manifestation of Mitochondrial Disorder (MID). The patient benefited from gabapentin and a variety of dietary supplements.

The presented patient is interesting for dysesthesia and dysautonomia attributed to SFN as a phenotypic manifestation of the mtDNA variant m.10191T>C in *MT-ND3*. Although SFN has been previously reported in MIDs other than due to m.10191T>C,^2^^,^^3^ it is a novel phenotypic trait of the m.10191T>C variant. SFN was attributed to the genetic defect since all causes of secondary SFN could be largely ruled out.

SFN generally affects the A-delta- and C-fibres and presents clinically, with pain, sensory disturbances, and autonomic dysfunction.^4^ Pain can occur in a heterogenous distribution, as acral neuropathic pain, in a stocking-like distribution, as focal or regional pain syndrome, or as whole-body pain. Sensory disturbances affect the protopathic modalities but not the epicritic sensitivity.^4^ All autonomic functions can be disturbed but SFN most commonly presents with POTS. Treatment of SFN is symptomatic and causal in some secondary type SFNs.

Mitochondrial SFN has only rarely been described so far. In a study of 27 MID patients using the sudoscan device, SFN was detected in 9.^2^ LFN was detected by NCSs in 11 of the 27.^2^ LFN was particularly detected in patients carrying *POLG1* variants, the 8344A>*G* variant, and the m.3243A>*G* variant.^2^ In patients with a single mtDNA deletion, LFN was detected in only 11%.^2^ Another patient, a 35-year-old male, complained of restlessness and had tingling in both feet for 2 years.^3^ A year before admission he developed burning toes and fingers bilaterally.^3^ Motor and sensory NCSs and F-responses were normal in the upper and lower limbs.^3^ Needle EMG was non-informative. On quantitative sensory threshold tests, cold thermal threshold and heat pain were above the 99th percentile, indicating hypoesthesia. Cardio-vagal autonomic tests assessing sympathetic and parasympathetic responses were normal. After 18 months of follow-up, no progression of symptoms was evident. Since the patient carried the variant m.3243A>*G*, after the exclusion of all differential diagnoses, SFN was attributed to the mtDNA mutation.

In summary, the case demonstrates that the m.10191T>C variant in *MT-ND3* can present phenotypically with SFN in the context of Leigh-like syndrome presenting as a multisystem MID. Symptomatic treatment of pain and dysautonomia along with supplements can provide long-term reduction and relief of pain and autonomic dysfunction and can significantly improve the overall quality of life.

## Declarations

Ethics approval: Was in accordance with ethical guidelines. The study was approved by the institutional review board.

Consent to participate: Was obtained from the patient.

Consent for publication: Was obtained from the patient.

Availability of data: All data are available from the corresponding author.

Code availability: Not applicable.

## Authors’ contributions

JF: Design, literature search, discussion, first draft, critical comments, final approval; SM: Literature search, discussion, critical comments, final approval.

## Funding sources

No funding was received.

## Declaration of Competing Interest

The author declares that the research was conducted in the absence of any commercial or financial relationships that could be construed as a potential conflict of interest.
